# Electropolymerization without an electric power supply

**DOI:** 10.1038/s42004-022-00682-8

**Published:** 2022-05-27

**Authors:** Suguru Iwai, Taichi Suzuki, Hiroki Sakagami, Kazuhiro Miyamoto, Zhenghao Chen, Mariko Konishi, Elena Villani, Naoki Shida, Ikuyoshi Tomita, Shinsuke Inagi

**Affiliations:** 1 Department of Chemical Science and Engineering, School of Materials and Chemical Technology, Tokyo Institute of Technology, 4259 Nagatsuta-cho, Midori-ku, Yokohama, Kanagawa 226-8502 Japan; 2 Department of Chemistry and Life Science, Yokohama National University, 79-5 Tokiwadai, Hodogaya-ku, Yokohama, Kanagawa 240-8501 Japan; 3 PRESTO, Japan Science and Technology Agency (JST), 4-1-8 Honcho, Kawaguchi, Saitama 332-0012 Japan

**Keywords:** Electrochemistry, Synthetic chemistry methodology, Sustainability

## Abstract

Electrifying synthesis is now a common slogan among synthetic chemists. In addition to the conventional two- or three-electrode systems that use batch-type cells, recent progress in organic electrochemical processes has been significant, including microflow electrochemical reactors, Li-ion battery-like technology, and bipolar electrochemistry. Herein we demonstrate an advanced electrosynthesis method without the application of electric power based on the concept of streaming potential-driven bipolar electrochemistry. As a proof-of-concept study, the electrochemical oxidative polymerization of aromatic monomers successfully yielded the corresponding polymer films on an electrode surface, which acted as an anode under the flow of electrolyte in a microchannel without an electric power supply.

## Abstract (web summary)

Electrochemical synthesis has become a powerful tool, but the need for an electric power supply has disadvantages when considering sustainable development goals. Here, a streaming potential generated by a laminar flow of electrolyte in a microchannel acts as a driving force for electrode reactions, enabling electrochemical oxidative polymerization of aromatic monomers without an electric power supply.

## Introduction

Electrochemistry has been a powerful tool for organic synthesis, and now a growing number of mainstream synthetic chemists are exploring the combination of traditional synthetic methodologies with electrochemistry, which has been termed “electrifying synthesis”^1^. Chemical reductants and oxidants are not necessary, but rather electrons from/to an electrical power source can be used for organic reduction and oxidation (redox) reactions, which also contributes in part to sustainable development goals (SDGs). The fine tuning of potentials applied to organic molecules is effective to result in reaction selectivity such as chemo-, regio- and pathway-selectivity^2–12^. In addition to the conventional two- or three-electrode systems that use batch-type cells, recently, there has been significant progress in organic electrochemical processes such as microflow electrochemical reactors^13–18^, Li-ion battery-like technology^19^ and bipolar electrochemistry^20^. It is obvious that electric current is supplied from an electric power source in electrolytic cells. However, if electrosynthetic reactors are free from dependence on power supplies, an ideal and ubiquitous elctrosynthesis system would be available in unpowered areas, including deep sea and aerospace.

**Fig. 1:**
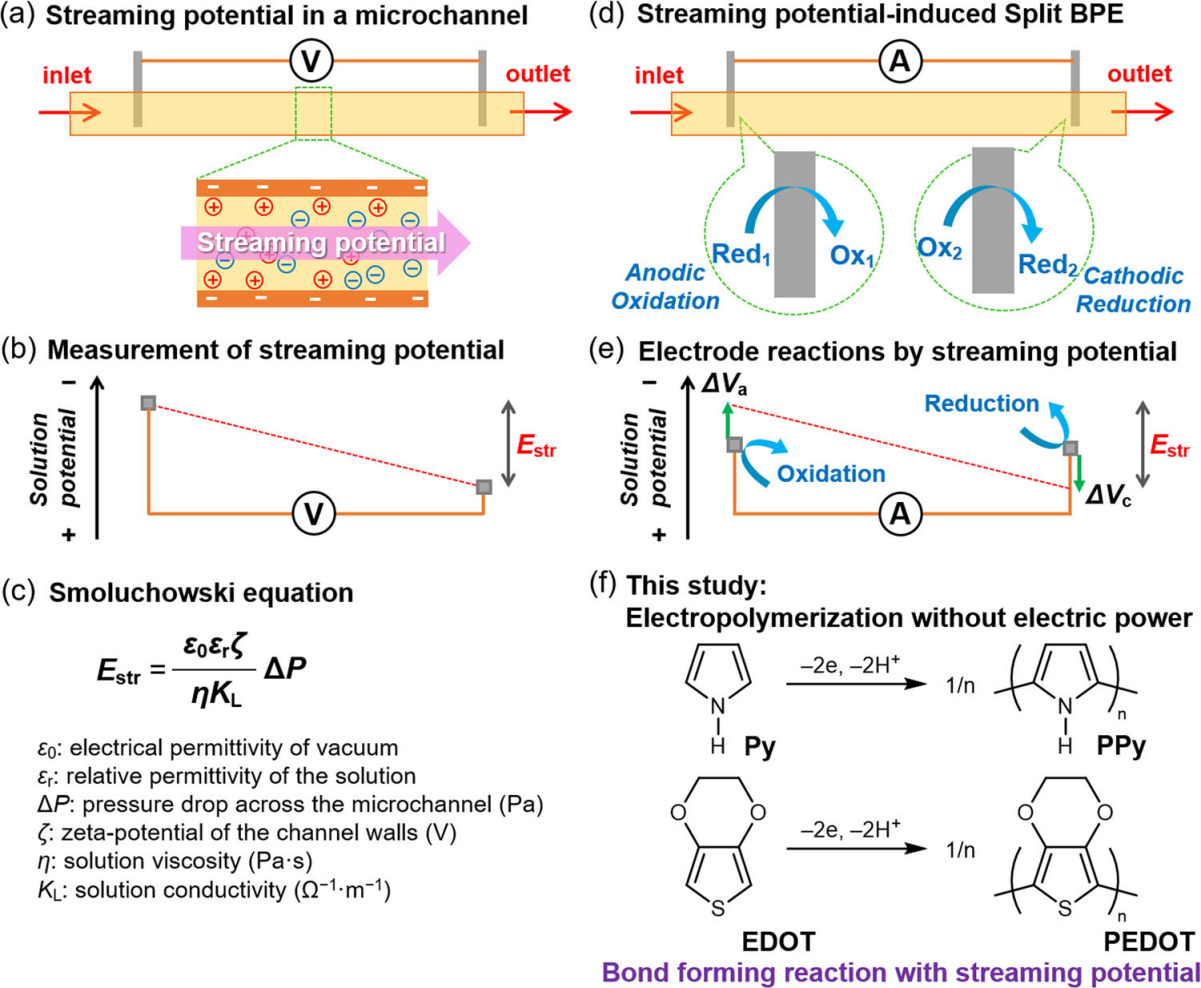
Conceptual illustration of this study. **a** The pressure-flow of electrolyte in a microchannel generates a streaming potential. **b** The streaming potential (*E*_str_) can be measured with a voltmeter connecting two probes. **c** Smoluchowski equation. **d** Split BPE is driven under the pressure-flow condition when two electrodes are connected with an ammeter (low resistance). **e** The split BPE can drive electrode reactions without an electric power supply. **f** This study demonstrates anodic electropolymerization of aromatic monomers, i.e., the first example of bond forming reactions, with streaming potential.

To solve such self-contradiction, the key is the use of a streaming potential generated by a laminar flow of electrolyte in a microchannel as a driving energy for electrode reactions, which is compatible with the development of flow electrochemical systems. Streaming potential (*E*_str_) originates from charge imbalance by a pressure gradient through a microchannel with charged walls (Fig. 1a–c). This is a class of electrokinetic phenomena that is often used in the analytical technique for zeta-potentials of materials and colloid surfaces^21^, but never involving reactions of any species in electrolytes. A pioneering work on electrode reactions based on streaming potentials was reported by Crooks et al. to demonstrate the anodic dissolution of metallic silver (Ag) inserted in a microchannel made of polysiloxane resin with the flow of an aqueous solution^22^. The motivation for the present study is to develop an electrosynthesis method without an electrical power supply to perform organic synthesis beyond analytical uses. As shown in Fig. 1d, e, two electrodes located at upstream and downstream under a streaming potential (connected each other with a conductive wire or a low resistance ammeter) are able to work as a split bipolar electrode (BPE) involving redox reactions^23^. According to the principle of bipolar electrochemistry^24–26^, when *E*_str_ (the difference of anodic and cathodic overpotentials, Δ*V*_a_ and Δ*V*_c_, respectively) is larger than the difference of anodic and cathodic potentials of redox species in a system, electrode reactions can occur on the split BPE. Therefore, the generation of a few volts of *E*_str_ enables redox reactions of organic molecules to occur at a split BPE for organic electrosynthesis. In this context, such a concept would realize electrochemical reactions producing value-added materials by simply passing a solution through a channel without an external power supply. In this proof-of-concept study, the electrochemical oxidative polymerization of aromatic monomers as an electro-organic reaction is demonstrated as the first example of bond forming reaction using streaming potentials (Fig. 1f).

## Results

### Streaming potential generation with organic electrolytic solutions

A custom-made plastic (polyether ether ketone; PEEK) cell, composed of two chambers with a platinum (Pt) wire and a PEEK microtube (0.50 mm inner diameter) connecting both chambers, was used for the following experiments (Supplementary Fig. 1). Since the microtube is simply connected between the two chambers, it can be replaced as needed when the filling material deteriorates to maintain performance reproducibility. According to the principle of streaming potential (i.e., the Smoluchowski equation, Fig. 1c)^21^, streaming potential (*E*_str_) is proportional to pressure drop (Δ*P*). To generate a pressure drop in a microtube, cotton wool was tightly filled inside the tube (see Methods). Since the downstream chamber is at atmospheric pressure, the value of pressure displayed by the pump reflects the pressure drop inside the microchannel. Therefore, Δ*P* can be monitored and tuned with a feeding pump by changing the flow rate (Supplementary Fig. 2). It should be noted that the channel interior must be composed of non-conductive materials, based on the principle of streaming potential.

**Fig. 2:**
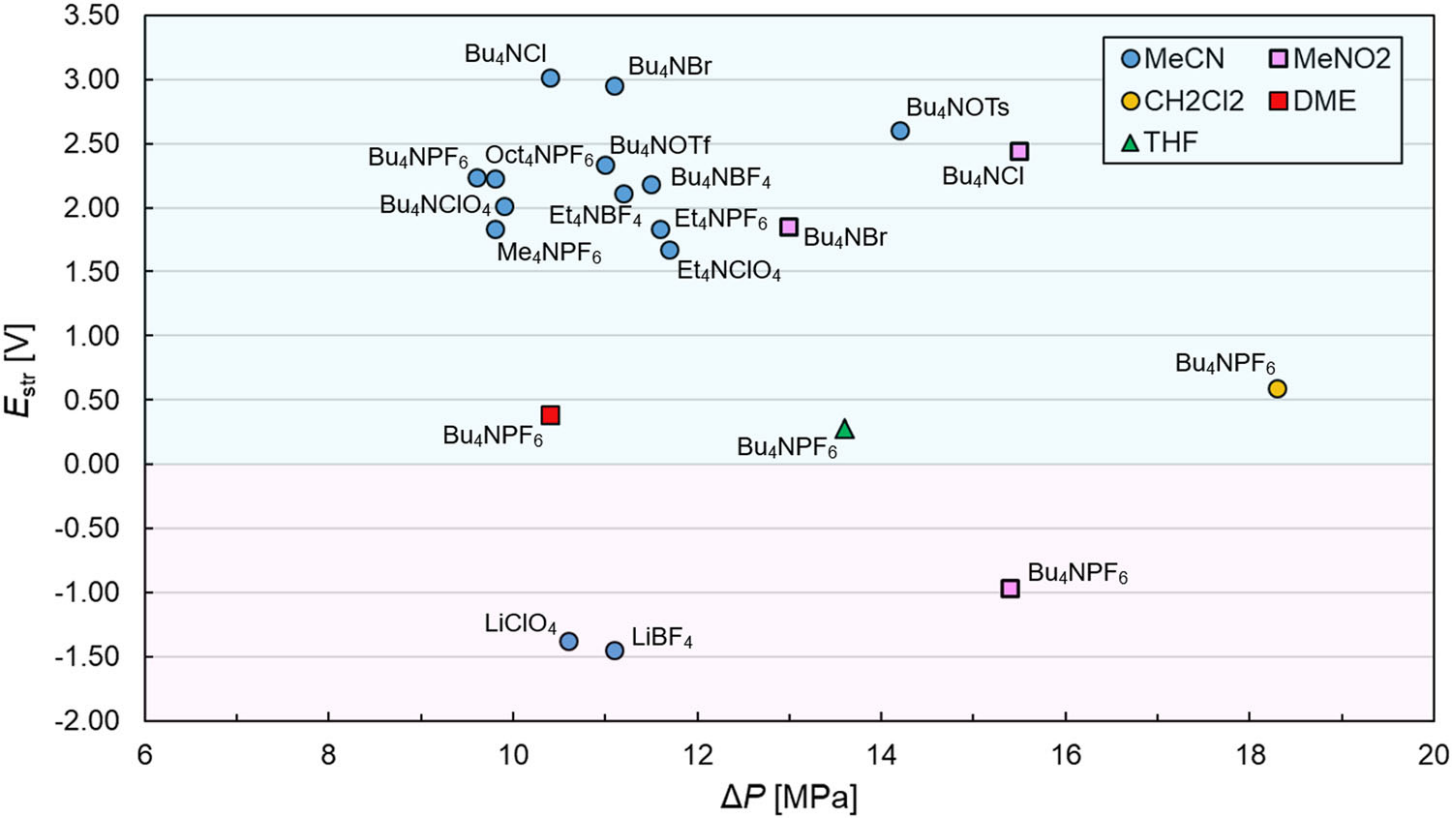
Map of streaming potential in each solution. Relationship between generated Δ*P* and observed *E*_str_ when various electrolytes containing 0.5 mM salts and organic solvents (blue circle: MeCN, yellow circle: CH_2_Cl_2_, green triangle: THF, pink square: MeNO_2_, red square: DME) are supplied at a flow rate of 0.5 mL/min.

The two Pt wires inserted inside the chambers were connected with a voltmeter to detect the *E*_str_ generated between the chambers by the flow of electrolyte (Supplementary Fig. 1b, c). A pure acetonitrile (MeCN) and an MeCN solution containing tetrabutylammonium hexafluorophosphate (Bu_4_NPF_6_) were initially examined for the measurement. A 0.5 mL/min flow rate of MeCN (10.1 MPa) resulted in *E*_str_ of 1.84 V. Even in the case of 0.5 mM Bu_4_NPF_6_/MeCN, a similar *E*_str_ (2.10 V, 8.8 MPa) was generated. The observed *E*_str_ gradually decreased by increasing the electrolyte concentration (Supplementary Fig. 3). Our aim is to use this system for the generation of organic ions and radical ions for subsequent reactions; therefore, a certain amount of supporting electrolyte (0.5 mM) was added to compensate the generated ionic species in the following experiments. The proportional relationship between *E*_str_ and Δ*P* (and flow rate) is shown in Supplementary Fig. 4. Considering the balance of conditions from these results, a flow rate of 0.5 mL/min was selected as a standard input parameter. The choice of solvent and electrolyte was very critical on the resultant streaming potential behavior. Streaming potentials generated in a 0.5 mM solution of Bu_4_NPF_6_ were highly dependent on the solvents used, i.e., *E*_str_ = 0.39 V (10.4 MPa) in dimethoxyethane (DME), 0.59 V (18.3 MPa) in dichloromethane (CH_2_Cl_2_), 0.28 V (13.6 MPa) in tetrahydrofuran (THF). In the case of nitromethane (MeNO_2_), a negative *E*_str_ was detected (−0.97 V with 15.4 MPa), which suggests that the opposite polarity is expected for a split BPE system. The *E*_str_ value was also highly dependent on the electrolyte, e.g., a 0.5 mM solution of LiBF_4_/MeCN showed −1.45 V (11.1 MPa) (Supplementary Fig. 5). An MeCN solution containing lithium hexafluorophosphate (LiPF_6_) also showed a negative streaming potential. However, Δ*P* gradually increased with a continuous flow. This is presumably due to deposition of lithium fluoride (LiF) in a microchannel^27^. The relationship between Δ*P* and *E*_str_ for various combinations of electrolytes and solvents is mapped in Fig. 2. According to the Smoluchowski equation, the value and polarity of *E*_str_ should be related to the zeta-potential of the microchannel material surface (mainly cotton wool) between the two electrodes; however, there is little library of zeta-potentials for materials in organic electrolytic solutions, unlike that for aqueous systems^28^. In addition, *E*_str_ also depends on permittivity, viscosity and conductivity of solutions, which are complex influences. Therefore, a map of *E*_str_ generated in different solvents (Fig. 2) is convenient for selecting electrolyte/solvent combinations in electrosynthesis applications below.

### Electropolymerization of aromatic monomers with streaming potentials

**Fig. 3:**
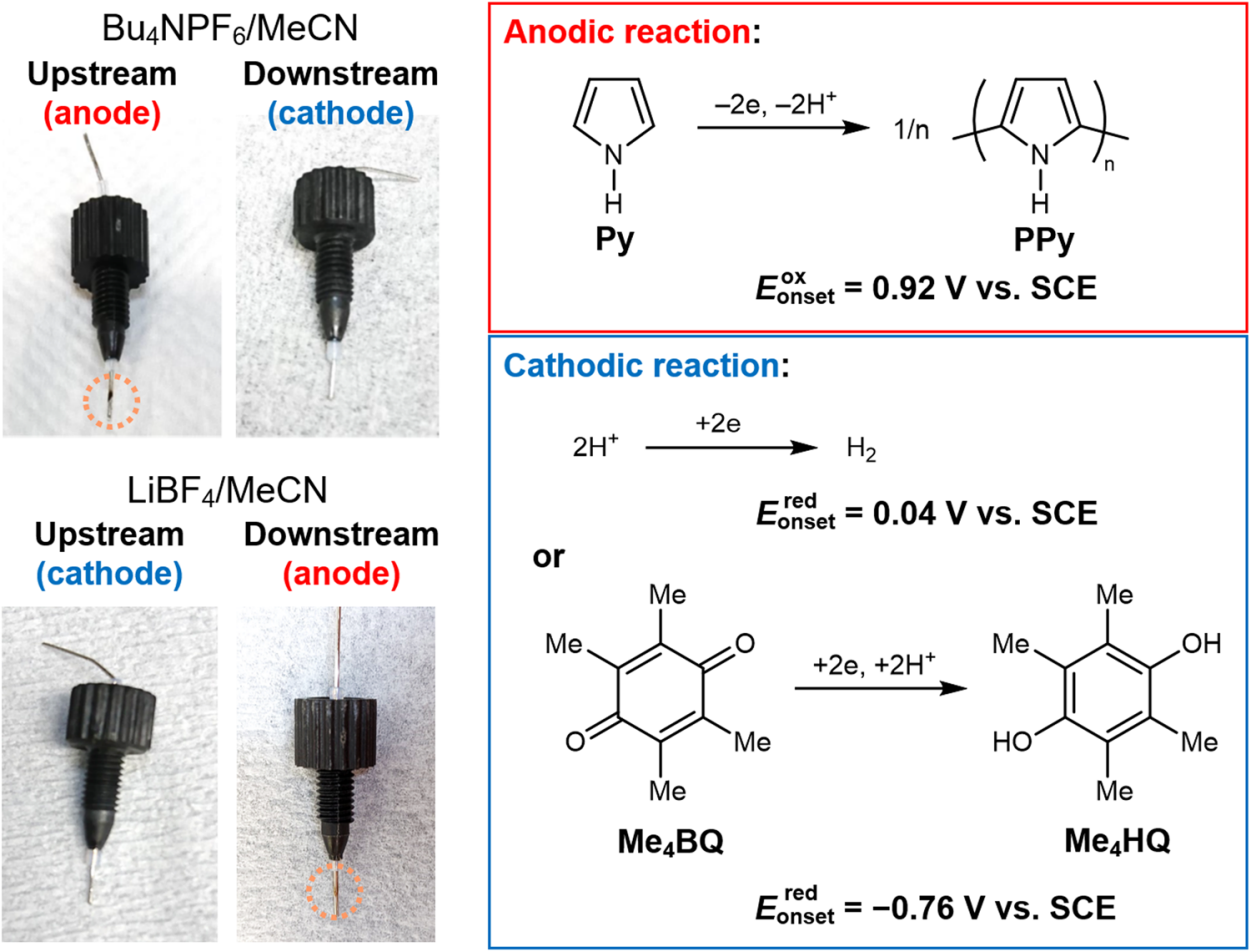
Electropolymerization of Py at the split BPEs. Photographs of the electrodes used in upstream and downstream chambers for electropolymerization of Py under different electrolyte conditions. Expected anodic and cathodic reactions are also included with their onset potentials observed in linear sweep voltammetry measurements.

From these results, organic electrosynthesis with a streaming potential was investigated under a flow condition with 0.5 mM Bu_4_NPF_6_/MeCN. To easily detect electrode reactions, the electropolymerization of aromatic monomers was selected, where one-electron oxidation occurs at an anode surface, followed by coupling and deprotonation, which results in the deposition of a conducting polymer film on the anode at the crossing position with the microchannel (see Supplementary Fig. 1d). A pyrrole monomer (Py) was used for the anodic reaction, i.e., oxidative electropolymerization, and proton reduction for cathodic reaction (Fig. 3). The number of electrons and protons for the reactions is balanced in the redox system. From linear sweep voltammograms of pyrrole and proton (*E*_onset_^ox^ = 0.92 V vs. SCE and *E*_onset_^red^ = 0.04 V vs. SCE, respectively), the threshold potential difference for the redox reactions (Δ*V*_BPE_) was estimated as ca. 0.88 V (Supplementary Fig. 6), which was less enough than the generated streaming potential. The two electrodes were connected to each other with an ammeter to drive a split BPE system, in which these redox reactions were triggered by a streaming potential (Fig. 3). Therefore, the faradaic current involved in a BPE can be monitored during the redox reactions^29–31^. Indeed, during flow of the electrolytic solution at 0.5 mL/min, an almost constant current (ca. 0.2 μA) was monitored with the ammeter. After continuous flow for 2 h, the upstream electrode was covered with polypyrrole (PPy) (Table 1, entry 1), whereas the downstream electrode was not covered at all (Fig. 3). This observation demonstrated that the former electrode worked as an anode, while the latter as a cathode of the split BPE, as expected from the polarity observed in the streaming potential measurement. Even considering a slower flow rate that could generate a streaming potential (1.08 V) just above the threshold (Δ*V*_BPE_ = 0.88 V), similar total charge and PPy deposition were observed after 50 mL of electrolyte was supplied (entry 2). Moreover, although a Py/MeCN solution without electrolytes generated relatively high *E*_str_ value as shown in Supplementary Fig. 3, PPy was not obtained on the electrode presumably because oxidation of Py hardly occurred due to the lack of anionic species to stabilize the oxidized species of Py (entry 3). As control experiments, (i) a pressure flow of the same electrolyte without connecting two electrodes (independent wires) resulted in no deposition of polymer film (entry 4), (ii) the split BPE system without Py monomer did not yield a deposition on the electrode (entry 5), (iii) neither Δ*P* nor current were generated in the absence of cotton wool (entry 6). However, even in the absence of Py monomer, a non-negligible current was detected during operation (entry 5), which is derived from redox reactions of the electrolytic solution and/or the streaming current^32,33^. Since the incremental current value in the presence of monomer was very small, it is difficult to clearly distinguish the faradaic current and discuss the faradaic efficiency of electropolymerization.

**Table 1:** Electropolymerization of Py monomers under different conditions.


Entry	Deviation from optimal conditions	Δ*P* [MPa]^a^	Current [μA]	Charge passed [mC]^b^	Polymer deposition
1	–	11.6	0.21	1.40	Upstream
2	Lower flow rate (0.25 mL/min)	7.3	0.10	1.37	Upstream
3	Without electrolyte	10.9	0.11	0.74	–
4	Without connection between electrodes	13.4	–	–	–
5	Without Py	10.1	0.21	1.36	–
6	Without cotton filling	0.0	0.00	0.00	–

^a^Pressure drop. ^b^50 mL of electrolytic solution was supplied.

With the positive results of streaming potential-driven electropolymerization of Py in hands, we next examined the effects of additives and electrolytes on the electrolytic reaction (Table 2). To assist cathodic electron transfer, 2,3,5,6-tetramethyl-1,4-benzoquinone (Me_4_BQ) was added as a sacrificial reagent for reduction, where its reduction onset potential *E*_onset_^red^ was −0.76 V vs. SCE and thus Δ*V*_BPE_ = 1.68 V (Supplementary Fig. 6). Although 1,4-benzoquinone (BQ) is often used as a sacrificial reagent for cathodic reduction, we chose Me_4_BQ as an additive in this experiment because reactivity of BQ with Py in an electrochemical process was reported^34^. However, there was little difference in the current observed and the amount of polymer film (entry 1).

**Table 2:** Results of electropolymerization of Py and EDOT monomers under different electrolyte conditions.

Entry^a^	Monomer (2.5 mM)^c^	Additive (0.25 mM)^d^	Electrolyte (0.5 mM)	Δ*P* [MPa]^e^	Current [μA]	Charge passed [mC]	Polymer deposition
1	Py	Me_4_BQ	Bu_4_NPF_6_/MeCN	15.0	0.23	1.495	Upstream
2	Py	Me_4_BQ	LiBF_4_/MeCN	7.6	−0.16	1.033	Downstream
3^b^	Py	Me_4_BQ	Bu_4_NPF_6_/MeNO_2_	15.7	−0.08	0.655	Downstream
4	EDOT	BQ	Bu_4_NPF_6_/MeCN	16.2	0.23	1.497	Upstream
5	EDOT	BQ	LiBF_4_/MeCN	10.0	−0.16	1.055	Downstream

^a^Flow rate: 0.5 mL/min. ^b^Flow rate: 0.4 mL/min. ^c^Py: pyrrole, EDOT: 3,4-ethylenedioxythiophene. ^d^Me_4_BQ: 2,3,5,6-tetramethyl-1,4-benzoquinone; BQ: 1,4-benzoquinone. ^e^Pressure drop.

To expect a polarity switch of electrodes, electropolymerization of Py monomer with 0.5 mM LiBF_4_/MeCN was carried out. It showed a negative current in operation, evidencing that a downstream electrode worked as an anode. Indeed, the downstream electrode was successfully covered with a PPy film (Table 2, entry 2). In this case, it was effective to add Me_4_BQ for enhancing the main cathodic reaction at the upstream electrode. Similarly, the Bu_4_NPF_6_/MeNO_2_ system afforded a negative current and film deposition on a downstream electrode (entry 3). It was determined that the sequence of target electrode reactions (oxidation/reduction or reduction/oxidation) can be controlled by the polarity of the streaming potentials.

Another monomer for oxidative electropolymerization was employed successively. 3,4-Ethylenedioxythiophene (EDOT) is a precursor for a promising conducting polymer, poly(3,4-ethylenedioxythiophene) (PEDOT), in a wide variety of fields of materials science. The combination of EDOT (*E*_onset_^ox^ = 1.31 V vs. SCE) and BQ as a sacrificial reagent for cathodic reduction (*E*_onset_^red^ = −0.42 V vs. SCE) was successful in this trial (Δ*V*_BPE_ = 1.73 V) (Supplementary Fig. 7). Electropolymerization of EDOT at both the upstream and downstream electrodes was demonstrated using 0.5 mM Bu_4_NPF_6_/MeCN and 0.5 mM LiBF_4_/MeCN, respectively (Table 2, entries 4 and 5).

### Characterization and properties of the conducting polymers

**Fig. 4:**
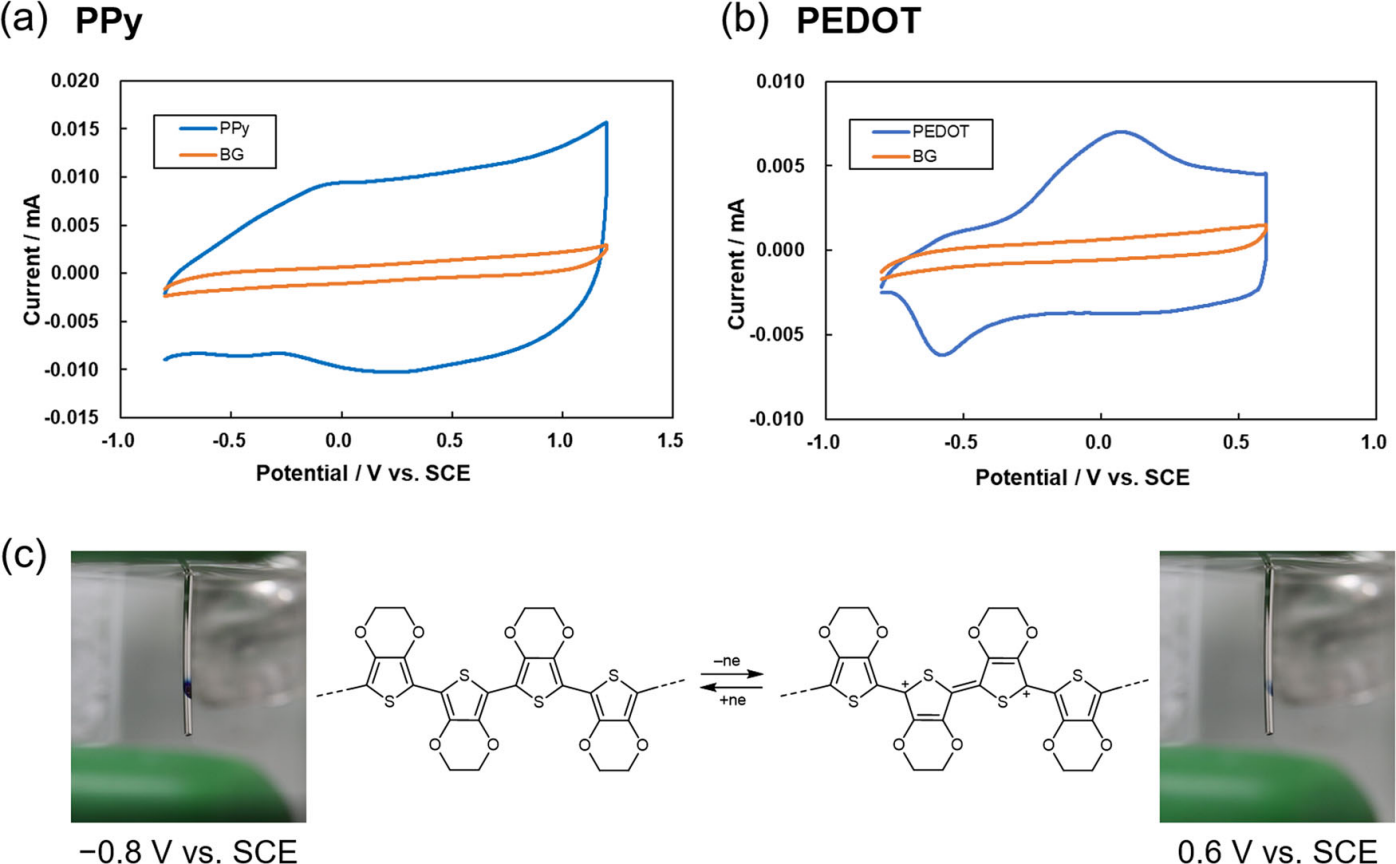
Properties of the obtained polymers. Cyclic voltammograms for (**a**) PPy and (**b**) PEDOT obtained on Pt electrodes by the streaming potential-driven electropolymerization detailed in Table 1, measured in 100 mM Bu_4_NPF_6_/MeCN at a scan rate of 0.1 V/s. **c** Photographs of the PEDOT film on the Pt electrode, showing electrochromic behavior during anodic potential scan.

The polymer films were characterized electrochemically using cyclic voltammetry. A Pt electrode with polymer deposition was used as a working electrode in a 100 mM Bu_4_NPF_6_/MeCN solution. Both PPy and PEDOT exhibited typical redox responses with the electrochemical doping/dedoping process. In particular, PEDOT showed a clear electrochromic property, i.e., color change from deep blue to colorless during the oxidation process, in which the absorption of the doped state (bipolaron) of PEDOT disappeared at visible light region (Fig. 4)^35^. Scanning electron microscopy (SEM) analysis of the obtained polymer films was also conducted. Both PPy and PEDOT films were very thin having a smooth surface regardless of the polarity of the streming potential (Supplementary Fig. 8). Compared to the polymer films obtained by conventional constant current electropolymerization of Py and EDOT monomers, the redox behavior and surface morphologies were very similar to those of the polymer films obtained in the present study (Supplementary Fig. 9).

## Discussion

In conclusion, a pressure-driven electrosynthetic reactor that does not require the application of electric power has been developed. A cotton-filled PEEK channel induced a streaming potential between two electrodes inside each chamber at the upstream and downstream of the electrochemical flow cell. The appropriate combination of electrolyte/organic solvent and flow conditions generated 2–3 V of streaming potential, which was sufficient to involve redox reactions of organic species. Electropolymerization of aromatic monomers such as Py and EDOT was successfully achieved with this system to afford the corresponding conducting polymer films on the electrodes at the upstream or downstream depending on the polarity of the streaming potential. This is the proof-of-concept study demonstrating anodic aromatic coupling reaction (electropolymerization) using streaming potentials. There are still several challenges to make this technology more practical, such as generating higher streaming potentials with lower pressure drop, and increasing the faradaic current compared to the streaming current. However, such electrosynthesis method without an electric power supply is quite a new concept toward environmentally friendly methodology for syntheses, also considering that only very low concentration of electrolyte is necessary. This process would also provide many possibilities in application to other electrochemical devices.

## Methods

### General considerations

All reagents and solvents were obtained from commercial sources and used without further purification. Platinum (Pt) wires were purchased from the Nilaco Corporation. Streaming potentials and current between a split bipolar electrode (BPE) was measured on a Sanwa PC773 multimeter recorded with a PC Link 7 software. Cyclic voltammetry (CV) and linear sweep voltammetry (LSV) measurements were carried out with an ALS 600 C Electrochemical Analyzer. CV measurements were conducted in a three-electrode system composed of a Pt working electrode (*ϕ* = 0.6 mm) with polypyrrole or poly(3,4-ethylenedioxythiophene) deposition, a Pt plate counter electrode (10 mm × 10 mm) and a saturated calomel electrode (SCE) as a reference electrode. LSV measurements were conducted in a three-electrode system equipped with a Pt disk working electrode (*ϕ* = 3 mm), a Pt plate counter electrode (10 mm × 10 mm) and a SCE as a reference electrode. Scanning electron microscopy (SEM) observation was conducted using a JEOL JSM 6610. Constant current electrolysis was carried out with a Hokuto Denko HABF-501A.

### Apparatus

A custom-made plastic (polyether ether ketone, PEEK) cell, composed of two chambers with a platinum (Pt) wire (*ϕ* = 0.6 mm) and a PEEK microtube (outer diameter: 1/16 inch) connecting both chambers, was used. The Pt wires were inserted inside the chambers with PTFE tubes. An electrolytic solution was fed by a pump with a constant feed rate. The two Pt wires inserted into the chambers were connected with a voltmeter or an ammeter (Supplementary Fig. 1).

### Optimization of flow conditions

Since the downstream chamber is at atmospheric pressure, the value of pressure displayed by the pump reflects the pressure drop (Δ*P*) inside the microchannel. Therefore, Δ*P* can be monitored and tuned with a feeding pump by changing the flow rate. Since bare PEEK tubes (inner diameters: 0.25 mm, 0.50 mm, 1.00 mm) did not generate Δ*P* at all, a cotton wool was tightly filled into the PEEK tubes. Considering the reproducibility of the measurements, 0.5 mm of inner diameter was selected as a standard with a filling of cotton wool (ca. 6 mg). Supplementary Fig. 2 shows the relationship between monitored Δ*P* and flow rate of an acetonitrile (MeCN) solution containing 0.5 mM tetrabutylammonium hexafluorophosphate (Bu_4_NPF_6_).

### Streaming potential measurements

Two Pt wires inserted into the chambers were connected with a voltmeter to detect a streaming potential (*E*_str_) generated in between the chambers by a flow of an electrolyte. A pure MeCN and MeCN solutions containing Bu_4_NPF_6_ were examined for the measurement. The observed *E*_str_ gradually decreased with increasing electrolyte concentration (Supplementary Fig. 3). The proportional relationship between *E*_str_ and Δ*P* (and flow rate) is shown in Supplementary Fig. 4. The dependency of *E*_str_ on concentration of LiBF_4_ in MeCN solutions was also plotted in Supplementary Fig. 5.

### Linear sweep voltammograms

To estimate the threshold potential difference for the redox reactions (Δ*V*_BPE_), linear sweep voltammetry was carried out with a three-electrode system (Supplementary Figs. 6 and 7). Δ*V*_BPE_ was calculated as the difference between onset oxidation potential (*E*_onset_^ox^) and onset reduction potential (*E*_onset_^red^).

### Electropolymerization with streaming potentials

Two Pt wires inserted into the chambers were connected with an ammeter to drive a split BPE for oxidative electropolymerization of aromatic monomers. During flow of the electrolytic solution at 0.5 mL/min, an almost constant current was monitored with the ammeter. After continuous flow for 2 h, the upstream electrode or downstream electrode was covered with a film of conducting polymers.

### Electropolymerization of Py with the conventional constant current method

A Pt wire and a Pt plate (10 mm × 10 mm) was used as an anode and a cathode, respectively, in 0.5 mM or 100 mM Bu_4_NPF_6_/MeCN electrolyte, and constant current electrolysis (0.23 μA) was carried out for 2 h.

## Supplementary information

Supplementary Information

## Data Availability

The data that support the findings of this study are available from the authors on reasonable request.
