# Supplementary material for: Electropolymerization without an electric power supply
Source: Commun Chem. 2022 May 27;5:66. doi: 10.1038/s42004-022-00682-8 (PMC9814265; doi:10.1038/s42004-022-00682-8)
Supplement: Supplementary file 1 — Supplementary Information [file 42004_2022_682_MOESM1_ESM.pdf]

Supplementary Information

Electropolymerization without an Electric Power Supply

Suguru Iwai,[1] Taichi Suzuki,[1] Hiroki Sakagami,[1] Kazuhiro Miyamoto,[1] Zhenghao Chen,[1] Mariko Konishi,[1] Elena Villani,[1] Naoki Shida,[1,2] Ikuyoshi Tomita,[1] and Shinsuke Inagi[*,1,3]*

[1]Department of Chemical Science and Engineering, School of Materials and Chemical Technology, Tokyo Institute of Technology, 4259 Nagatsuta-cho, Midori-ku, Yokohama, Kanagawa 226-8502, Japan

[2]Department of Chemistry and Life Science, Yokohama National University, 79-5 Tokiwadai, Hodogaya-ku, Yokohama, Kanagawa 240-8501, Japan

[3]PRESTO, Japan Science and Technology Agency (JST), 4-1-8 Honcho, Kawaguchi, Saitama 332-0012, Japan

E-mail: inagi@cap.mac.titech.ac.jp

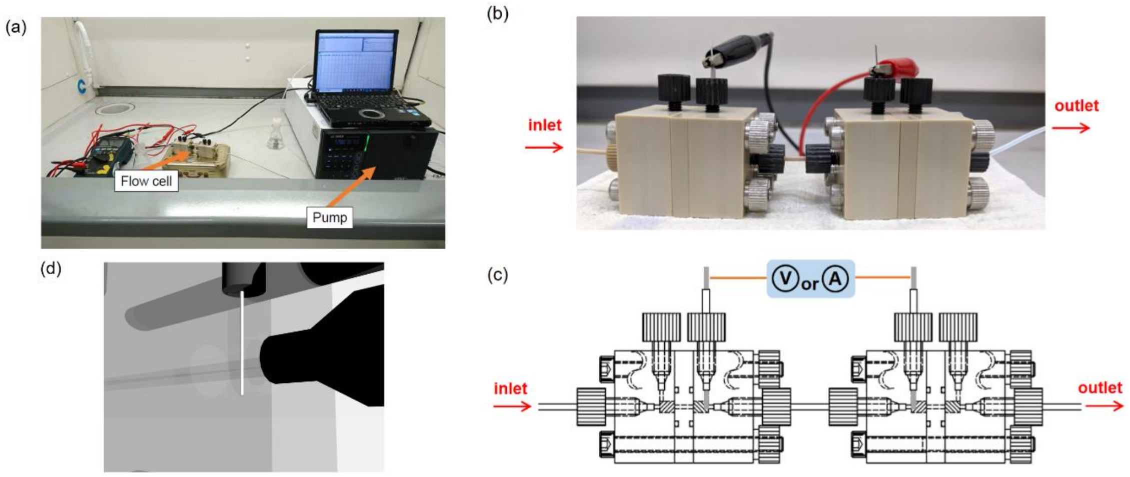

Supplementary Fig. 1. (a) A photographic image of the experimental setup composed of a feeder pump, cell units and a multimeter monitored by a PC-link software. (b),(c),(d) a photograph and design drawings of the cell chambers.

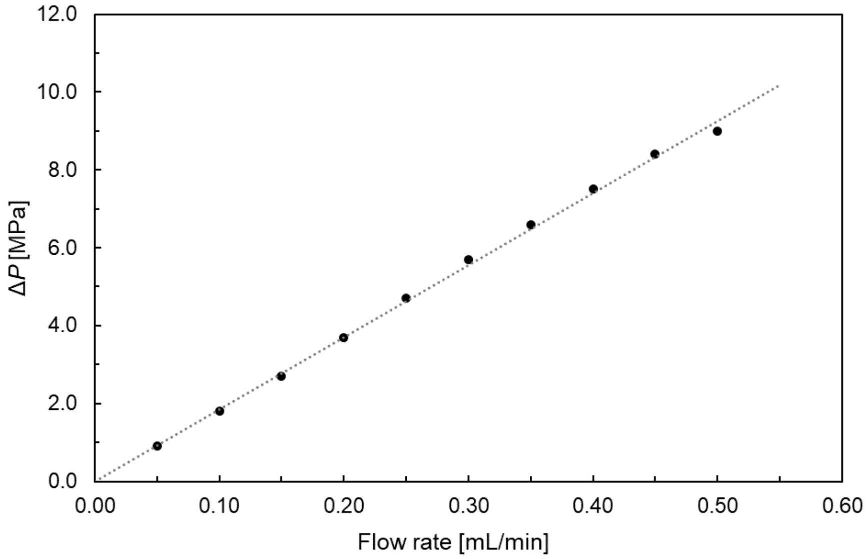

Supplementary Fig. 2. The data plot of Δ*P* and flow rate using 0.5 mM Bu_4NPF_6/MeCN.

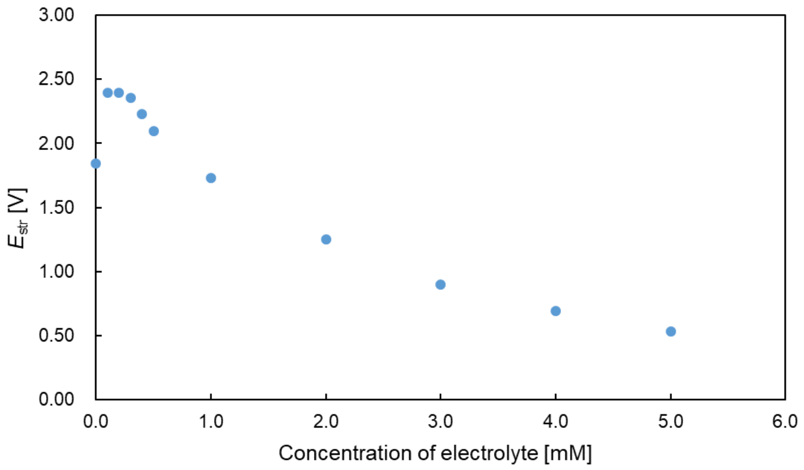

Supplementary Fig. 3. The dependency of E_{str} on concentration of Bu_4NPF_6 in MeCN solution fed at a flow rate of 0.5 mL/min.

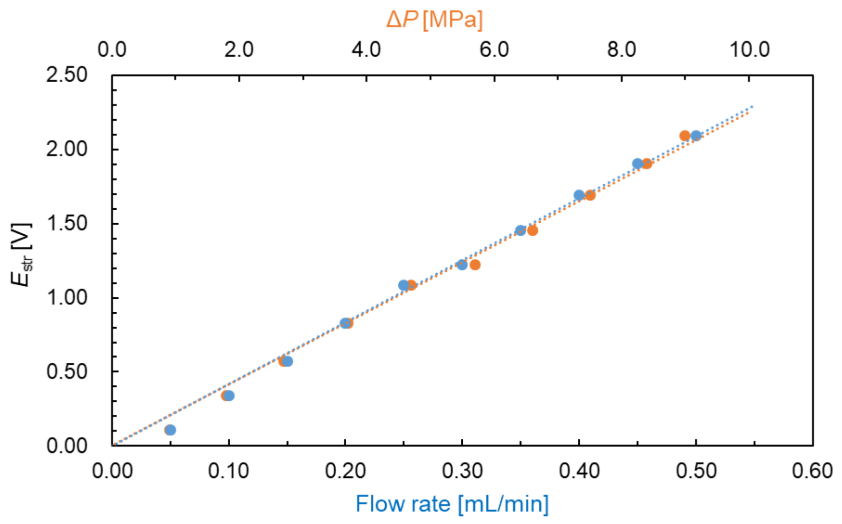

Supplementary Fig. 4. The linear plots of E_{str} and ΔP (and flow rate) using 0.5 mM Bu_4NPF_6/MeCN.

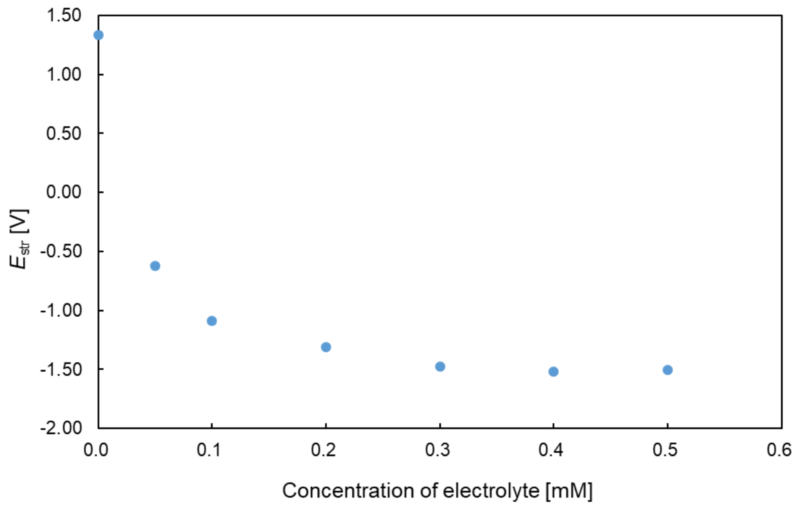

Supplementary Fig. 5. The dependency of E_{str} on concentration of $LiBF_4$ in MeCN solution fed at a flow rate of 0.5 mL/min.

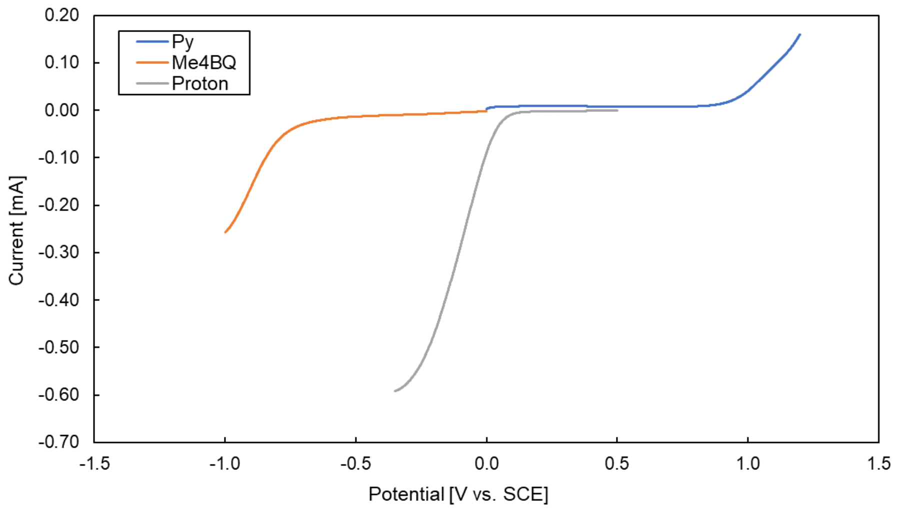

Supplementary Fig. 6. Linear sweep voltammograms of pyrrole (Py) for oxidation, and 2,3,5,6-tetramethyl-1,4-benzoquinone (Me_4BQ) and trifluoromethanesulfonic acid (indicated as Proton in the legend) for reduction with a Pt disk working electrode, measured in 100 mM Bu_4NPF_6/MeCN at a scan rate of 0.1 V/sec.

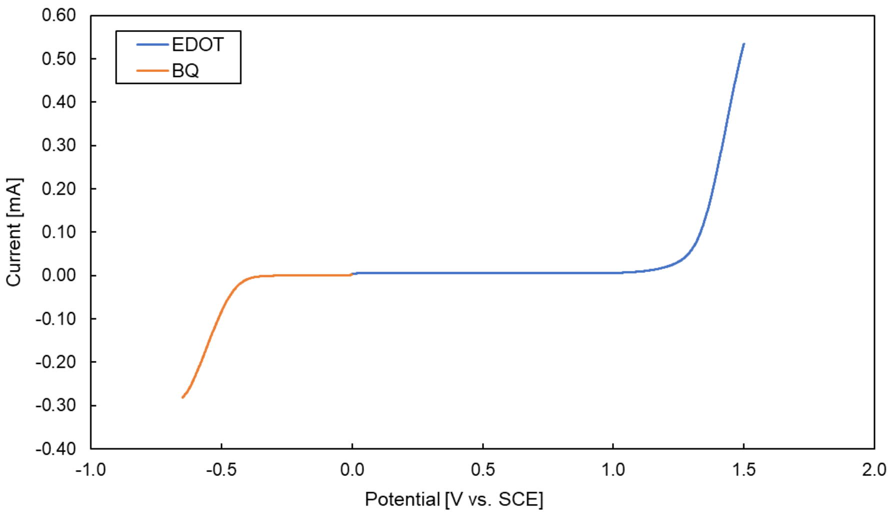

Supplementary Fig. 7. Linear sweep voltammograms of 3,4-ethylenedioxythiophene (EDOT) for oxidation and 1,4-benzoquinone (BQ) for reduction with a Pt disk working electrode, measured in 100 mM Bu_4NPF_6/MeCN at a scan rate of 0.1 V/sec.

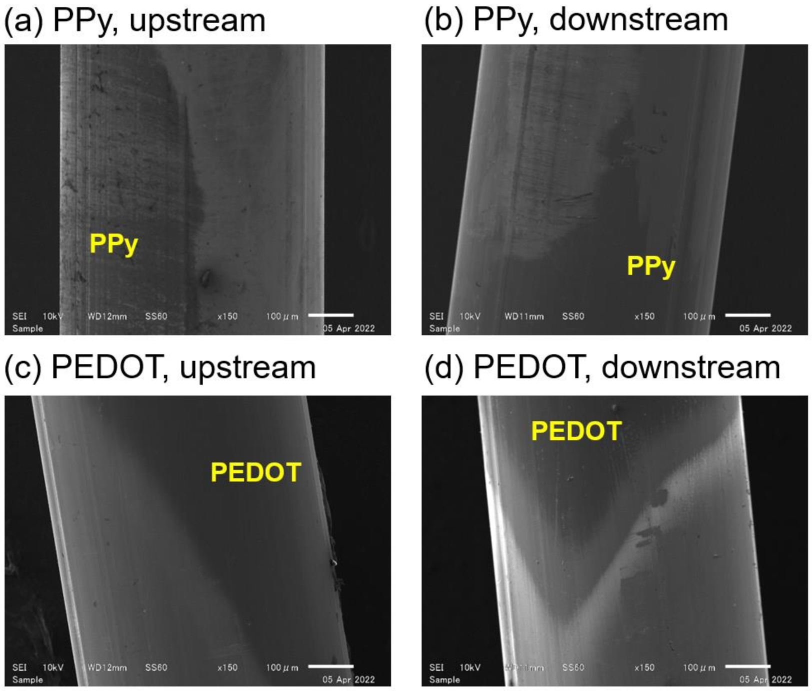

Supplementary Fig. 8. SEM images of conducting polymer films obtained on the Pt wire electrode by streaming potential method. (a) PPy at the upstream electrode, (b) PPy at the downstream electrode, (c) PEDOT at the upstream electrode, (d) PEDOT at the downstream electrode.

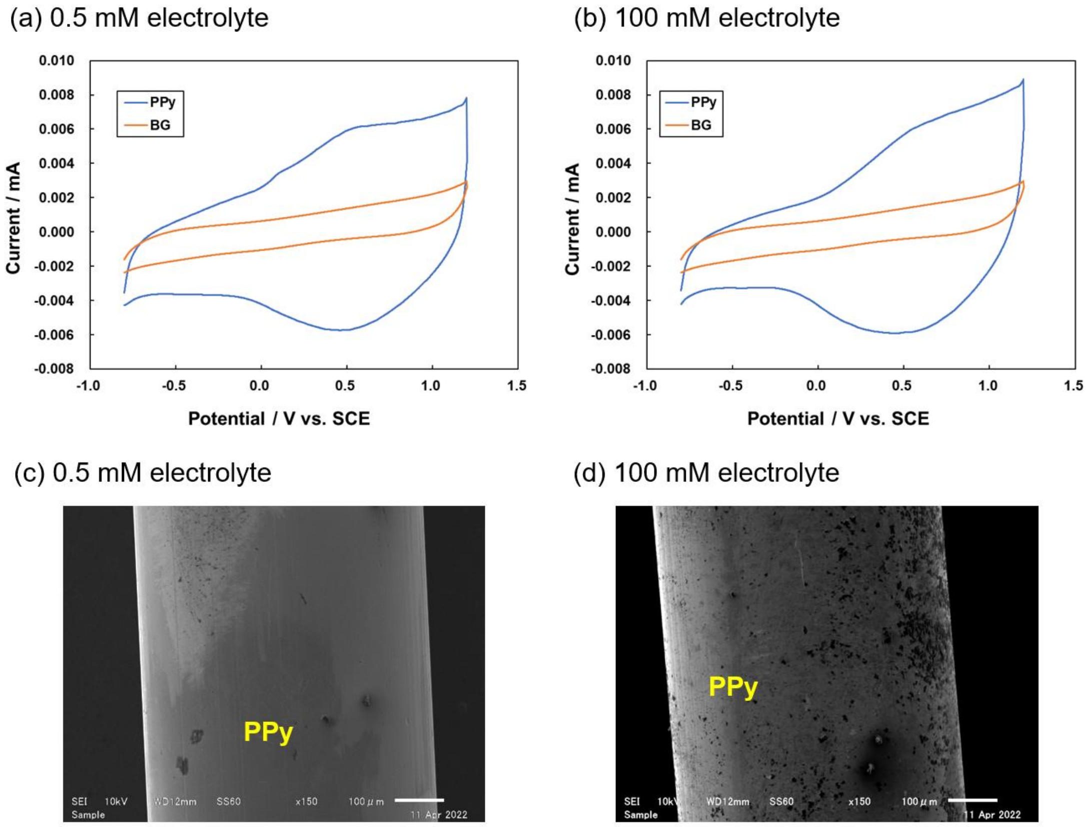

Supplementary Fig. 9. The experimental data of PPy prepared by conventional constant-current electropolymerization of Py monomer with different electrolyte concentrations (0.5 mM and 100 mM of Bu_4NPF_6/MeCN). (a,c) CV and SEM image of the PPy film obtained with 0.5 mM electrolyte concentration. (b,d) CV and SEM image of the PPy film obtained with 100 mM electrolyte concentration. CVs were measured using the polymer-coated Pt wire as a working electrode in 100 mM Bu_4NPF_6/MeCN at a scan rate of 0.1 V/sec.
